# Zearalenone Exposure Triggered Cecal Physical Barrier Injury through the TGF-β1/Smads Signaling Pathway in Weaned Piglets

**DOI:** 10.3390/toxins13120902

**Published:** 2021-12-15

**Authors:** Pengfei Zhang, Changwei Jing, Ming Liang, Shuzhen Jiang, Libo Huang, Ning Jiao, Yang Li, Weiren Yang

**Affiliations:** 1 Department of Animal Sciences and Technology, Shandong Agricultural University, Tai’an 271018, China; zhangpengfei@sdau.edu.cn (P.Z.); szjiang@sdau.edu.cn (S.J.); huanglibo@sdau.edu.cn (L.H.); jiaoning@sdau.edu.cn (N.J.); 2 Technical Department, Shandong Chinwhiz Co., Ltd., Weifang 262400, China; jingchangwei@chinwhiz.com; 3 Department of Feeding Microecology, Shandong Baolaililai Bioengineering Co., Ltd., Tai’an 271001, China; liangming@blll1004.onexmail.com

**Keywords:** zearalenone, cecum, physical barrier, TGF-β1/Smads; piglet

## Abstract

This study aims to investigate the effects of exposure to different dosages of zearalenone (ZEA) on cecal physical barrier functions and its mechanisms based on the TGF-β1/Smads signaling pathway in weaned piglets. Thirty-two weaned piglets were allotted to four groups and fed a basal diet supplemented with ZEA at 0, 0.15, 1.5, and 3.0 mg/kg, respectively. The results showed that 1.5 and 3.0 mg/kg ZEA damaged cecum morphology and microvilli, and changed distribution and shape of M cells. Moreover, 1.5 and 3.0 mg/kg ZEA decreased numbers of goblet cells, the expressions of TFF3 and tight junction proteins, and inhibited the TGF-β1/Smads signaling pathway. Interestingly, the 0.15 mg/kg ZEA had no significant effect on cecal physical barrier functions but decreased the expressions of Smad3, p-Smad3 and Smad7. Our study suggests that high-dose ZEA exposure impairs cecal physical barrier functions through inhibiting the TGF-β1/Smads signaling pathway, but low-dose ZEA had no significant effect on cecum morphology and integrity through inhibiting the expression of smad7. These findings provide a scientific basis for helping people explore how to reduce the toxicity of ZEA in feeds.

## 1. Introduction

Zearalenone (ZEA), a toxic mycotoxin usually produced by Fusarium species, is found in cereal grains and derived products such as maize and barley [1,2,3]. Due to the presence of ZEA, there are plenty of crops lost per year. Additionally, ZEA poses a hidden threat to health of humans and livestock through the food chain [4]. Previous study suggested that ZEA compete for estrogen receptor sites and contribute to reproduction problems, which even can result in genital organ alterations of pigs resulting in disorders of the hormonal balance of the body [5]. In addition to its negative effect on the reproductive system, ZEA also has hepatotoxicity, immunotoxicity, hematotoxicity, and genotoxicity [2]. When ZEA is introduced into animals and humans in the diet, they first come to interact with the gastrointestinal tract. Therefore, researchers are increasingly interested in the effects of ZEA on gut health [4,6].

The intestinal epithelium is the initial line of defense against external harmful microbes and plays an important role in preventing the entry of toxicity of ZEA [7]. When animals and humans consume contaminated feed, their intestinal epithelial cells are immediately exposed to ZEA, leading to intestinal barrier injury. Many studies have shown that exposure to ZEA could impair gut morphology, increase intestinal permeability, and decrease the expression of tight junction proteins (TJs) in the small intestine [6,8]. The cecum, which is important in the intestine of pig, absorbs water and electrolytes, and provides a controllable route both for the excretion of waste products obtained through metabolism and for toxic substances. In addition, the cecum contains abundant bacterial flora which can ferment nutrients to volatile fatty acids [9,10]. Crucially, the physical barrier of the cecum hinders the invasion of microorganism [11,12]. Previous studies in pigs have reported that after exposure to ZEA, the number of goblet cells in the cecum significantly decreased, suggesting that ZEA exposure might lead to cecal injury [13]. However, little literature is available to evaluate the effects of dietary ZEA exposure on cecal physical barrier functions, and the pathway by which ZEA damages the cecum is also unclear.

Transforming growth factor-β (TGF-β), a multifunctional cell factor, has functions of regulation in gut mucosal cell growth, differentiation, migration, and epithelial restitution [14,15]. Previous work has demonstrated that TGF-β1 was vital in the restoration of barrier integrity in vitro [16,17,18]. The Smad proteins are a class of molecules that mediate and modulate TGF-β family members [19]. The main Smads involved in this pathway are Smad2, Smad3, Smad4, and Smad7. The Smads convey their signals from TGF-β receptors in the cell surface into the nucleus, regulating the expression of target genes [20]. So far, there has been no research regarding the effects of ZEA on the TGF-β1/Smads signaling pathway in the cecum of piglets.

The present study was conducted to assess the impact of exposure to different dosages of ZEA on cecal physical barrier functions and to explore whether ZEA supplementation could affect cecal injury restoration through the TGF-β1/Smads signaling pathway.

## 2. Results

### 2.1. Effect of ZEA Exposure on Cecal Histomorphology in Pigs

**Figure 1:**
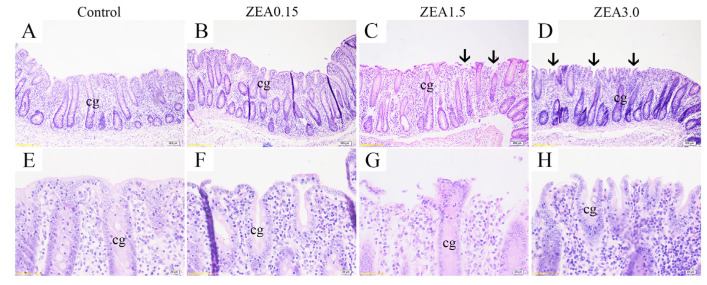
Light micrograph of cecal section in pig. Panels (**E**–**H**) (×400 magnification) were enlarged respectively from (**A**–**D**) (×100 magnification) showing enlarged structure of cecal glands (cg). Control, ZEA0.15, ZEA1.5, and ZEA3.0 were basal diet supplemented with 0, 0.15, 1.5, and 3 mg/kg of zearalenone (ZEA). Scale bars were 100 µm for (**A**–**D**), 20 µm for (**E**–**H**). cg represents cecal gland or cecal crypt.

The cecal morphological structure is presented in Figure 1. In the Control (Figure 1A,E), the cecal mucosa were smooth and intact. The intact cecal glands were neatly distributed in the mucosal layer. Occasional epithelial abscission was observed in the ZEA0.15 treatment group (Figure 1B,F). The ZEA1.5 treatment (Figure 1C,G) group showed severely damaged mucosa and broken epithelia of the cecal glands (black arrow). In the ZEA3.0 treatment group (Figure 1D,H), large areas of the mucosal epithelium were exfoliated and the damage to the mucosal epithelium extended deeper into the mucosal layer. In short, damage to the cecal mucosa became more and more serious with increasing ZEA concentration.

### 2.2. Effect of ZEA Exposure on Cecal Ultrastructure in Pigs

**Figure 2:**
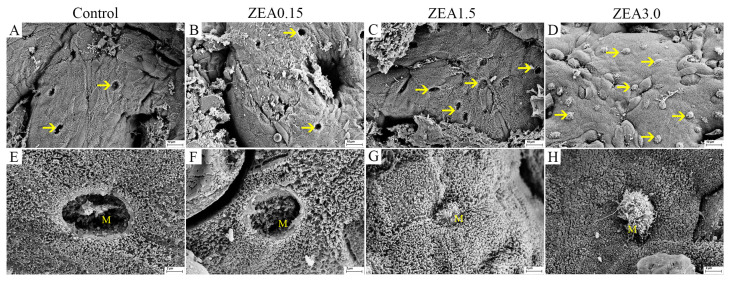
Electron micrographs of cecal mucosal epithelia in pig. Control, ZEA0.15, ZEA1.5, ZEA3.0 were basal diet supplemented with 0, 0.15, 1.5 and 3 mg/kg of ZEA. (**A**–**D**) ×1000 magnifications of the mucosal epithelium to observe the distribution of M cells (yellow arrows). Scale bar = 10 μm. (**E**–**H**) ×5000 magnification of whole M cell to observe details of M cell in all treatments. Scale bar = 2 μm. The yellow M represents microfold cells.

The M cells (yellow arrow) were more frequently found in the ZEA1.5 (Figure 2C) and ZEA3.0 (Figure 2D) treatments of ZEA than in the Control (Figure 2A) and ZEA0.15 treatments (Figure 2B). The microvilli of epidermal cells were damaged in the ZEA1.5 (Figure 2G) and ZEA3.0 (Figure 2H) treatments. Meanwhile, the M cells presented convex shape in the ZEA1.5 treatment, and the shape was more convex observed in the ZEA3.0 treatment.

### 2.3. Effect of ZEA Exposure on Distribution and Number of Goblet Cells in the Cecum of Pigs

**Figure 3:**
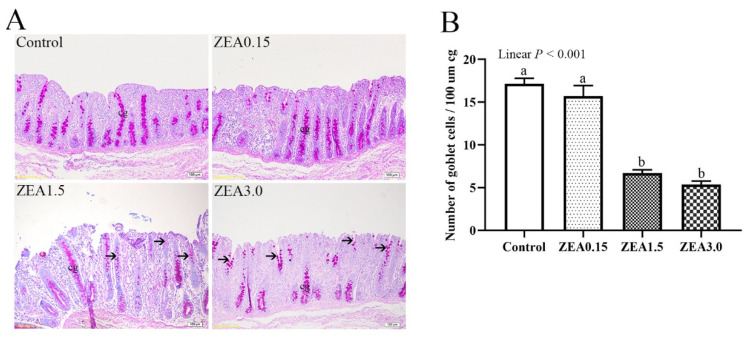
(**A**) The distribution of goblet cells in pig cecum section through PAS staining. (**B**) Number of goblet cells in cecum. Control, ZEA0.15, ZEA1.5, and ZEA3.0 represent the control diet with an addition of 0, 0.15, 1.5, and 3.0 mg/kg ZEA, respectively. The number of goblet cells was calculated per 100 μm of gland length separately for each cecal gland (cg). ^a, b^ Means differ significantly (*p* < 0.05). The cg represents cecal gland or cecal crypt.

Changes in goblet cells induced by ZEA are presented in Figure 3A. Goblet cells were mainly located in the epithelial cells of the cecal glands. The goblet cells were evenly and tightly distributed from the deep layer to the shallow layer of the lamina propria in the Control and ZEA0.15 groups. The distribution of goblet cells in the dehisced cecal glands was sparse in the ZEA1.5 treatment group, especially the ZEA3.0 treatment group.

The number of goblet cells per 100 μm of gland length (Figure 3B) decreased linearly (*p* < 0.001) with an increasing levels of ZEA. The pigs in ZEA1.5 and ZEA3.0 treatments showed significantly decreased numbers of goblet cells (*p* < 0.05) compared with the Control. There was no significant difference between the ZEA0.15 and the Control treatments (*p* > 0.05).

### 2.4. Effect of ZEA Exposure on Distribution and Expression of TFF3 in the Cecum of Pigs

**Figure 4:**
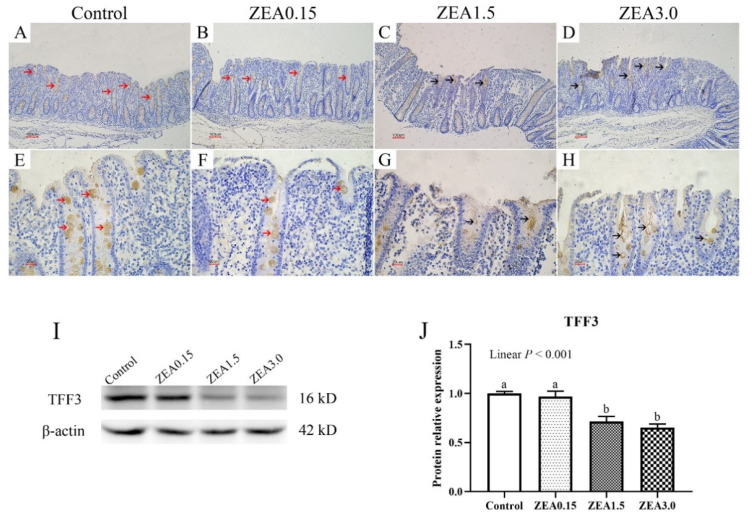
Trefoil factor family 3 (TFF3) localization in the pig cecum (**A**–**H**). Panels (**E**–**H**) (×400 magnification) were enlarged from (**A**–**D**) (×100 magnification) showing enlarged cecal glands to observe immunoreactive cells. Scale bars were 100 µm for (**A**–**D**), 20 µm for (**E**–**I**), protein bands of TFF3 and β-actin in cecum. (**J**), the protein levels of TFF3 in cecum, which were normalized to β-Actin. Control, ZEA0.15, ZEA1.5, and ZEA3.0 represent the control diet with an addition of 0, 0.15, 1.5, and 3.0 mg/kg ZEA, respectively. ^a, b^ Means differ significantly (*p* < 0.05).

Immunohistochemical analysis showed that a light brown granular immunoreactive substance of TFF3 was mainly localized in the goblet cells in the cecal glands of pigs (Figure 4A–H). Immunoreactive cells of TFF3 were densely distributed in the epithelium of the cecal glands and the light brown immunoreactive substances were evenly distributed in goblet cells of pigs in the Control (Figure 4A,E, red arrow) However, the distribution of immunoreactive cells that localized in the epithelium of damaged cecal glands was sparse and the immunoreactive substances were unevenly distributed in goblet cells in the ZEA1.5 (Figure 4C,G, black arrow) and ZEA3.0 treatments (Figure 4D,H), with even no immunoreactive cells in one entire cecal gland in the ZEA3.0 treatment group. There was no significant difference between the ZEA0.15 treatment (Figure 4B,F) and the Control.

We further tested the protein levels of TFF3 (Figure 4I,J), and the results showed a similar trend as in immunohistochemical detection. The relative protein expression of TFF3 decreased linearly (*p* < 0.001) with an increasing level of ZEA. The additions of 1.5 and 3.0 mg/kg ZEA in the piglet diet significantly inhibited TFF3 protein levels (*p* < 0.05).

### 2.5. Effect of ZEA on Expressions of Tight Junction Proteins in the Cecum of Pigs

**Figure 5:**
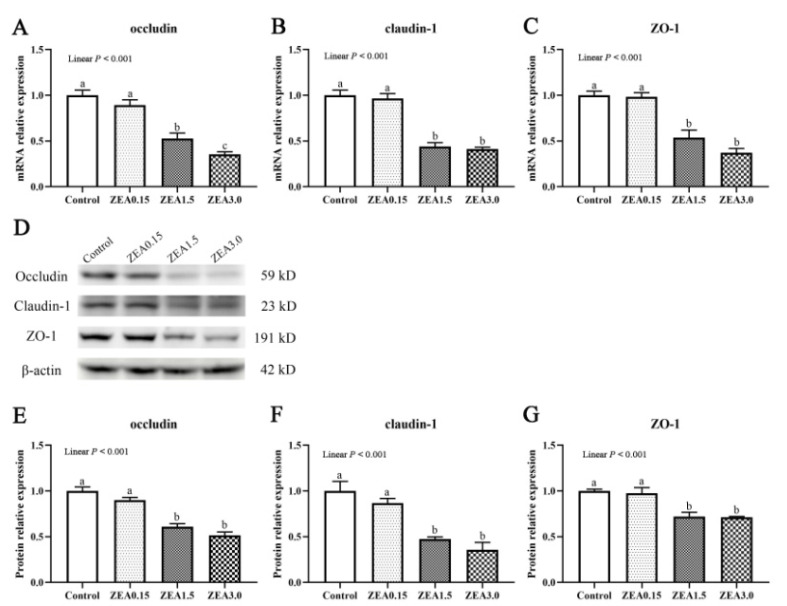
Expression of tight junction proteins in the cecum. (**A**–**C**), relative mRNA expressions of occludin, claudin-1, and ZO-1, as quantified by qPCR. (**D**–**G**) showed the western blotting results of the proteins occludin, claudin-1, and ZO-1 in the cecum. Quantified protein level was normalized to that of β-actin. Control, ZEA0.15, ZEA1.5, and ZEA3.0 were basal diet supplemented with 0, 0.15, 1.5, and 3 mg/kg, respectively, of ZEA. ZO-1, zonula occluden-1. ^a, b, c^ Means differ significantly (*p* < 0.05).

The mRNA expressions of tight junction proteins in the cecum are shown in Figure 5A–C. The relative mRNA expressions of occludin, claudin-1, and ZO-1 decreased linearly (*p* < 0.001) with an increasing level of ZEA. The mRNA expressions of occludin, claudin-1, and ZO-1 in ZEA1.5 and ZEA3.0 treatments were significantly lower than those in the Control and ZEA0.15 treatments (*p* < 0.05). In addition, pigs in ZEA3.0 treatment showed significantly lower occludin mRNA expression than pigs in ZEA1.5 treatment (*p* < 0.05).

The protein expressions of occludin, claudin-1, and ZO-1 were also determined in the present study (Figure 5D–G). Pigs in the Control and ZEA0.15 groups had significantly higher protein expressions of occludin, claudin-1, and ZO-1 than pigs in the ZEA1.5 and ZEA3.0 groups (*p* < 0.05).

### 2.6. Effect of ZEA on the TGF-β1/Smads Signaling Pathway in Cecum of Pigs

**Figure 6:**
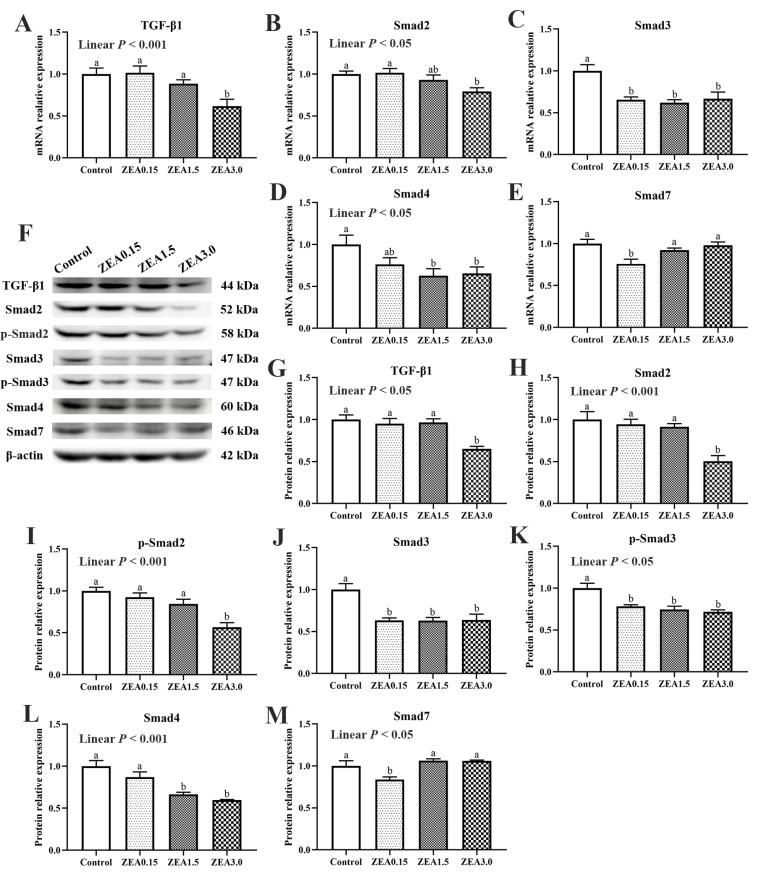
(**A**–**E**), mRNA relative expression of TGF-β1, Smad2, Smad3, Smad4, and Smad7 in the cecum. (**F**–**M**) show the Western blotting results of the proteins TGF-β1, Smad2, p-Smad2, Smad3, p-Smad3, Smad4, and Smad7 in the cecum. Quantified protein level was normalized to that β-actin. Control, ZEA0.15, ZEA1.5, and ZEA3.0 were basal diet supplemented with 0, 0.15, 1.5, and 3 mg/kg, respectively, of ZEA. TGF-β1, transforming growth factor-β1. ^a, b^ Means differ significantly (*p* < 0.05).

The mRNA expression of TGF-β1, Smad2, Smad3, Smad4, and Smad7 in the cecum are shown in Figure 6A–E. The relative mRNA expressions of TGF-β1, Smad2, and Smad4 decreased linearly (*p* < 0.05) with increasing ZEA concentration in the diet. The mRNA expression of TGF-β1 in the ZEA3.0 treatment group was obviously lower than that in the Control, ZEA0.15, and ZEA1.5 treatments (*p* < 0.05). The mRNA expression of Smad2 in the ZEA3.0 treatment group was obviously lower than that in the Control and ZEA0.15 treatments (*p* < 0.05). Compared with the Control, the pigs in the ZEA0.15, ZEA1.5, and ZEA3.0 treatments showed significantly decreased mRNA expression of Smad3 (*p* < 0.05). The mRNA expression of Smad4 in the ZEA1.5 and ZEA3.0 treatments were significantly lower than those in the Control and ZEA0.15 treatments (*p* < 0.05). The mRNA expression of Smad7 in the ZEA0.15 treatment was significantly lower than that in the Control, ZEA0.15, and ZEA3.0 treatments (*p* < 0.05).

The protein expressions of TGF-β1, Smad2, p-Smad2, Smad3, p-Smad3, Smad4, and Smad7 were also determined in the present study (Figure 6F–M). Different concentrations of ZEA had inconsistent effects on the protein expressions of TGF-β1 and Smads. The protein levels of TGF-β1, Smad2, and p-Smad2 in the ZEA3.0 treatment group were obviously lower than those in the Control, ZEA0.15, and ZEA1.5 treatment groups (*p* < 0.05). The protein levels of Smad3 and p-Smad3 in the ZEA0.15, ZEA1.5, and ZEA3.0 treatments significantly decreased (*p* < 0.05) compared with the Control treatment. The protein expressions of Smad4 in the ZEA1.5 and ZEA3.0 treatments were obviously lower than those in the Control and ZEA0.15 treatments (*p* < 0.05). The protein expression of Smad7 in the ZEA0.15 treatment was significantly lower than that in the Control, ZEA1.5, and ZEA3.0 treatments (*p* < 0.05).

## 3. Discussion

The intestinal epithelium is the initial line of defense against external harmful microbes and toxins from feeds and foods [7]. The gut epithelial cells, mucous layer, and TJs situated at the membrane junction provide the structural foundation of the physical barrier [21]. After ingestion of ZEA-contaminated food or feed, ZEA will cause adverse effects on the intestines. In our study, treatment with 0.15 mg/kg ZEA had a slight effect on cecal morphology, but 1.5 and 3.0 mg/kg of ZEA caused obvious damage to the epithelium of the mucosa and cecal gland. Furthermore, we found, by using scanning electron microscopy, that the distribution and pattern of M cells (microfold cells) on the mucosal epithelia of the cecum changed abnormally in the ZEA1.5 and ZEA3.0 groups, characterized by damaged and shortened microvilli of the epidermal cells and convex shapes of the M cells. The basolateral membrane of normal M cells presented invaginated “pockets” that house infiltrating lymphocytes [22]. M cells have a crucial function in the collection and transfer of antigens. Barrier functions and immune responses can be activated by M cells after the toxic stimuli from the microbes [23]. The changes of M cells in the present study indicated cecal mucosa layer injury, which might be due to the toxicity of ZEA shortening the microvilli of epidermal cells. In addition, the present study showed that the additions of 1.5 and 3.0 mg/kg ZEA in the piglet diet significantly decreased expressions of tight junction proteins (including occludin, claudin-1, and ZO-1), but there was no obvious change in pigs fed basal diet supplemented with 0.15 mg/kg of ZEA. Neighboring cells are sealed by tight junction proteins such as occludins, claudins and the zonula occludens proteins. Between endothelial and epithelial cells, tight junction proteins are important as a physical barrier which maintains intestinal integrity and separates toxins and pathogens from the internal intestinal environment [7,24]. Tight junction proteins are indispensable for attachment, and maintaining structure and biological function of epithelial cells [25]. Above all, the results of the present study showed that high-dose ZEA disrupted the cecal epithelial barrier by destroying the intestinal epithelial cells and mucosal layer and reducing the expressions of tight junction proteins.

Intestinal self-repair plays a vital role in inherently resisting severe damage. The surface of the intestinal wall is a single epithelial cell layer, and the goblet cell is among the single epithelial cells [26,27]. In addition, the mucus layer mainly consists of mucin, which is secreted by goblet cells [28,29]. Hence, goblet cells play a vital role in maintaining the physical barrier of the intestine. In the current study, the numbers of goblet cells in ZEA1.5 and ZEA3.0 treatments were significantly lower than those in the Control and ZEA0.15 treatments. Trefoil factor 3 (TFF3), which is also secreted by goblet cells to compose the cecal mucus barrier, is important for epithelial restoration [30]. Previous studies showed TFF3 promotes intestinal epithelial restoration as well as mucosal protection. Studies have shown the overexpression of TFF3 enhanced the resistance of rats to damage and ulceration of the intestine. After stimulation by dextran sodium sulfate, TFF3-deficient mice were more likely to suffer from colitis [31,32]. The pigs in ZEA1.5 and ZEA3.0 treatments showed significantly decreased protein levels of TFF3 compared with the Control. In addition, the distributions of TFF3 immunoreactive cells that localized in the epithelium of damaged cecal glands were sparse and the immunoreactive substances were unevenly distributed in goblet cells in the ZEA1.5 and ZEA3.0 treatments. We believe that ZEA damaged the goblet cells, which leaded to a decrease in TFF3 secretion. Therefore, we speculated that the damage of cecal epithelial barrier induced by high doses of ZEA was related to obstruction of intestinal self-repair.

In order to investigate the mechanism by which ZEA damaged the cecal epithelial barrier in our study, we determined the expression of the TGF-β1/Smads signaling pathway. TGF-β1 plays a vital role in intestinal injury restoration, and a majority of cytokines depend on TGF-β1 to facilitate epithelial restitution [33]. When the intestine is damaged, TGF-β1 facilitates epithelial cell migration and increases integrin and the extracellular matrix to improve intestinal epithelial and mucosal repair [17,18,34]. It was reported that TGF-β1 could improve the damaged intestinal epithelia, induced by interferon-γ, of infections of enterohemorrhagic *Escherichia coli* and *Cryptosporidium parvum* [16,17,18]. In addition, research in vitro also showed that TGF-β1 could alleviate the decreases in expressions of occludin and ZO-1 in IPEC-J2 induced by TNF-α [35]. The classical TGF-β1 signaling pathway is mediated by Smad family proteins. The main Smads involved in this pathway are Smad2, Smad3, Smad4, and Smad7, vital mediators of the TGF-β1 signaling transducer. After TGF-β1 stimulation, Smad 2 and Smad 3 require prior phosphorylation, which subsequently complex with Smad4. The complex then regulates gene expression by entering into the nucleus [20,36]. TGF signaling is inhibited by Smad7, which acts as a critical negative regulator [37,38]. In our study, the addition of 3.0 mg/kg of ZEA in the piglet diet significantly decreased the expression of TGF-β1, which might weaken cecal injury restoration. In addition, we found that expressions of Smad2, p-Smad2, Smad3, p-Smad3, and Smad4 in the ZEA3.0 treatment group decreased significantly. This demonstrated that the addition of 3.0 mg/kg ZEA in the piglet diet might hinder cecal epithelial restoration by directly inhibiting the expressions of TGF-β1 and downstream signals (Smad2, P-Smad2, Smad3, P-Smad3, and Smad4). In the ZEA1.5 treatment group, the expressions of Smad3, p-Smad3, and Smad4 decreased significantly though no significant change was observed in the expression of TGF-β1 compared with the control. Previous studies reported that, after TGF-β1 stimulation, Smad2, Smad3, and Smad4 form Smad2/Smad2/Smad4, Smad3/Smad3/Smad4 or Smad2/Smad3/Smad4 complexes. Smad4 plays an important role in these complexes and TGF-β signaling is substantially crippled in the absence of Smad4 [39]. Our study demonstrated that the addition of 1.5 mg/kg ZEA in the piglet diet may hinder cecal epithelial restoration by reducing the expressions of Smad3, p-Smad3, and Smad4. TGF-β induces intestinal epithelial cells to differentiate into goblet cells [40], and TFF3 is secreted by goblet cells [30]. In our study, the TGF-β1/Smads signaling pathway was inhibited in ZEA1.5 and ZEA3.0 treatments indicating that ZEA also indirectly restrained the formation of goblet cells and TFF3 by inhibiting the TGF-β1/Smads signaling pathway. Moreover, the expressions of Smad3, p-Smad3, and Smad7 in the ZEA0.15 treatment group decreased significantly, but the expressions of Smad2, p-Smad2, and Smad4 did not change significantly in our study. This might mean that the addition of 0.15 mg/kg ZEA in piglet diet did not severely inhibit the formation of the R-Smad–Smad4 complex. That is to say, the addition of 0.15 mg/kg ZEA in the piglet diet was not enough to cause serious damage to the cecum of pigs. As for the reason for the decreased expression of smad7, we guess it may be a sort of self-regulation of the body at low doses of ZEA, which needs further research.

## 4. Conclusions

In summary, ZEA could damage the cecal barrier through affecting the TGF-β1/Smads signaling pathway in a dose-dependent manner, resulting in changes of cecal morphology and goblet cells, as well as the expressions of TFF3 and TJs. Meanwhile, high concentrations of ZEA (1.5 and 3.0 mg/kg diet) impair cecal barrier integrity by decreasing the expressions of TFF3 and tight junction proteins through inhibiting the TGF-β1/Smads signaling pathway. However, in present study, a low concentration of ZEA (0.15 mg/kg diet) had no significant effect on cecal morphology and integrity through decreasing the expression of smad7, which might be attributed to a kind of self-regulation of the body. These findings give a solid foundation for future research into ways to reduce the toxicity of ZEA in feeds and foods.

## 5. Materials and Methods

### 5.1. Ethical Approval

The experimental procedures used in this study were reviewed and approved by the Institutional Animal Care and Use Committee of Shandong Agricultural University (Identification code: # SDAUA-2020-0710, Date of approval: 10 July 2020).

### 5.2. Experimental Design and Animal Management

Thirty-two Duroc × Landrace × Yorkshire weaned female piglets (35 d of age, 17.39 ± 0.24 kg), were randomly assigned to one of the following four dietary treatments (*n* = 8). (1) CON group (piglets were fed with a basal diet), (2) ZEA0.15 group (piglets were fed with the basal diet supplemented with 0.15 mg/kg ZEA), (3) ZEA1.5 group (piglets were fed with the basal diet supplemented with 1.5 mg/kg ZEA) and (4) ZEA3.0 group (piglets were fed with the basal diet supplemented with 3.0 mg/kg ZEA). The limit of ZEA in piglet feed in China is 0.15 mg/kg, and the other two doses were based on previous studies [41,42,43]. The treatment methods of purified crystalline ZEA, which were purchased from Fermentek (Jerusalem, Israel), are referred to in a previous study [44]. The basal diets used in the present study were formulated to meet or exceed the nutrient requirements recommended by the National Research Council (NRC, 2012) for weaned piglets and the ingredient composition and nutrient levels are shown in Supplementary Table S1. The time of the formal experiment was 32 days after 7-d adjustment. The trial location was at the Animal Research Station of Shandong Agricultural University (Tai’an, Shandong, China). During the entire trial period, piglets were fed ad libitum. The temperature of the pigsty was kept at 26–28 °C.

### 5.3. Mycotoxins Determination in Diet

The determination was completed by Qingdao Entry-Exit Inspection and Quarantine Bureau as described previously [44,45,46]. The methods for determining the concentrations of ZEA and aflatoxin (AFL) were liquid chromatography in conjunction with fluorescence detection, affinity column chromatography, and the external standard method. The levels of fumonisin (FUM) and deoxynivalenol (DON) were quantified using high-performance liquid chromatography tandem mass spectrometry with fluorescence detection, affinity column chromatography, and the external standard method [45,47]. The limit of detection (LOD) for ZEA, aflatoxin (AFL), fumonisin (FUM), and deoxynivalenol (DON) were 0.01 mg/kg, 1.0 μg/kg, 0.1 mg/kg, and 0.05 mg/kg, respectively. Six parallel samples from each diet were taken to determine the levels of ZEA. The analyzed ZEA were 0.14 ± 0.00, 1.45 ± 0.02, and 3.12 ± 0.07 mg/kg in ZEA0.15, ZEA1.5, and ZEA3.0 treatments. Determination results of ZEA in the control diet and other toxins were below the LOD.

### 5.4. Sample Collection

On the final day of the trial, the abdomen of the pig was immediately opened after intramuscular anesthesia and the gastrointestinal tract was removed to separate the cecum. A part of cecum was rinsed by physiological saline and fixed in 4% paraformaldehyde for 24 h for morphology, immunohistochemistry, and goblet cell examination. Another part of cecum sample was fixed in 2.5% glutaraldehyde for ultrastructural examination. In addition, the rest of the cecum samples were washed in ice-cold physiological saline solution, and immediately put into liquid nitrogen, followed by being stored at −80 °C until further analysis. 

### 5.5. Cecal Histology Examination

After fixation with 4% formaldehyde for 24 h, the cecum tissues were dehydrated in ethanol and xylene solutions followed by conventional embedding in paraffin. The embedded tissues of the cecum were cut into slices of 6 μm and a part of the histological sections mounted on poly-L-lysine-coated glass slides for goblet cell examination and immunohistochemical analysis. Then the sections were immersed in hematoxylin and eosin for the nuclear and cytoplasmic staining. Cecal structure was observed using an Olympus BX41 microscope equipped with a DP25 digital camera (Olympus, Tokyo, Japan).

### 5.6. Ultrastructural Examination

The cecal samples were fixed in a mixture of 1% paraformaldehyde and 2.5% glutaraldehyde in a 0.2 mol/L phosphate buffer. After being washed in PBS, the cecal samples were dipped in 2% osmium tetroxide for 12 h. Subsequently, the post fixed samples were washed, dehydrated, and then attached to aluminum stubs and coated with a thin layer of gold (sputtering method). The specimens were examined with a Carl Zeiss Geminisem300 (Oberkochen, Germany) Scanning Electron Microscope.

### 5.7. Identification and Examination of Goblet Cells

The PAS Stain kit (Nanjing Jiancheng Institute of Bioengineering, Nanjing, China) was used to stain cecal slides to identify goblet cells. Two slides were examined to determine the number of goblet cells by using an Olympus BX41 microscope equipped with a DP25 digital camera (Olympus, Tokyo, Japan). A total of 15 integrated, well-oriented cecal glands per sample were chosen to determine the number of goblet cells by counting goblet cells in both sides of the cecal gland. Due to the differences in the observed length of glands, we determined the mean number of goblet cells per 100 μm of gland length separately for each gland.

### 5.8. Immunohistochemistry Analysis

Immunohistochemistry detected distribution of TFF3. A commercial kit (Polink-2 plus^®^ Polymer HRP Detection system for rabbit primary antibody, PV-9001, ZSGB-BIO, Beijing, China) was used to perform immunohistochemical analysis. Sections were processed in accordance with the standard IHC protocols. The specific experimental procedure was carried out in strict accordance with the manufacturer’s instructions (details in Supplemental Methods), as previously described [44]. The localization of immunoreactive substances were observed by using an Optika B-510MET-R (Ponteranica, Italy) microscope equipped with a SC600 digital camera (Optika, Ponteranica, Italy).

### 5.9. Gene Expression

AG RNAex Pro (Accurate Biology, Changsha, China) was used to extract the total RNA of cecal samples. The cDNA was acquired by reverse transcription (RT) kit (Accurate Biology, Changsha, China). Quantitative real-time polymerase chain reaction (qRT-PCR) was performed to analyze the expression levels of TGF-β1, Smad2, Smad3, Smad4, and Smad7. The qRT-PCR reactions were performed on a LightCycler 96 (Roch, Switzerland) with SYBR^®^ Green Premix Pro Taq HS qPCR Kit (AG11701, Accurate Biology, DaLian, China) (details in Supplemental Methods), as previously described [44]. Gene primers are shown in Supplemental Table S2.

### 5.10. Western Blot Analysis

Protein expressions in cecal tissues were detected by Western blotting (operation details in Supplemental Methods), as previously described [44]. Primary antibodies against TFF3, occludin, claudin-1, ZO-1, TGF-β1, Smad2, Phospho-Smad2 (Ser465 + Ser467), Smad3, Phospho-Smad3 (Ser423 + Ser425), Smad4, and Smad7 were obtained from BIOSS (Beijing, China). Blots were quantified with Fusion 16.0.9.0 software (Vilber Lourmat, Paris, France).

### 5.11. Statistical Analysis 

The individual pig was used as the experimental unit. All data were analyzed by using the General Linear Model (GLM) in SAS 9.4 (SAS Institute Inc., Cary, NC, USA), and variations among the treatments were compared with Duncan’s multiple range tests. Results were presented as mean and standard error of the mean (SEM). Orthogonal polynomial contrasts were then used to determine linear responses to the ZEA levels of treatments. All statements of significance are based on a probability of *p* < 0.05.

## Data Availability

Not applicable.

## Supplementary Materials

The following are available online at https://www.mdpi.com/article/10.3390/toxins13120902/s1, Table S1: Ingredients and nutrient contents of the basal diet (air-dry basis), Table S2: Primers for relative-quantitative real-time PCR; Supplemental methods.
