# Supplementary material for: Zearalenone Exposure Triggered Cecal Physical Barrier Injury through the TGF-β1/Smads Signaling Pathway in Weaned Piglets
Source: Toxins (Basel). 2021 Dec 15;13(12):902. doi: 10.3390/toxins13120902 (PMC8708673; doi:10.3390/toxins13120902)
Supplement: Supplementary file 1 [file toxins-13-00902-s001.zip › toxins-1493913-supplementary.pdf]

Supplementary Materials: Zearalenone Exposure Triggered Cecal Physical Barrier Injury through the TGF-β1/Smads Signaling Pathway in Weaned Piglets

Pengfei Zhang, Changwei Jing, Ming Liang, Shuzhen Jiang, Libo Huang, Ning Jiao, Yang Li, and Weiren Yang

Table S1. Ingredients and nutrient contents of the basal diet (air-dry basis).

Ingredients	Content (%)	Nutrients	Analyzed Values
Expanded corn	64.43	ME，MJ/kg	13.86
Whey powder, CP 3%	5	Crude protein	18.48
Fermented soybean meal	14	Calcium	0.74
Expanded soybean	8.5	Total phosphorus	0.62
Fish meal	4	Available phosphorus	0.39
$CaHPO_4$	1.15	Lys	1.38
Pulverized Limestone	0.7	Met	0.4
Sodium chloride	0.2	Sulfur amino acid	0.66
L-Lysine HCl	0.76	Thr	0.85
DL-Met	0.08	Trp	0.23
L-Thr	0.16		
L-Trp	0.02		
Premix[1]	1		
Total	100		

[1] Supplied per kg of diet: VA, 2300 IU; VD_3, 230 IU; VE, 20 IU; VK_3, 0.60 mg; VB_1, 1.80 mg; VB_2, 4.25 mg; Pantothenic acid, 13.00 mg; Niacin, 20.00 mg; pyridoxine, 2.00mg; Biotin, 0.09 mg; Folic acid, 0.45 mg; VB_{12}, 0.020 mg; Mn (Manganese methionine), 4.00 mg; Fe (Iron (II) fumarate), 90 mg; Zn (Glycine zinc), 90 mg; Cu (bis(glycinato)copper), 6.00 mg; I (Calcium Iodate), 0.14 mg; Se (Seleniwm Yeast), 0.30 mg.

Table S2. Primers for relative-Quantitative real-time PCR.

Target Gene	Accession No.	Primer Sequence (5' to 3')	Product Size Bp
β-actin	XM_021086047.1	F: GGACTTCGAGCAGGAGATGG<br>R: AGGAAGGAGGGCTGGAAGAG	138
Occludin	NM_001163647.2	F: ACAGATCCACGAGCAGCAAA<br>R: TGGCAATGAACACCATCACA	175
Claudin 1	NM_001244539.1	F: TCAATACAGGAGGGAAGCCAT<br>R: ATATTTAAGGACCGCCCTCTCC	90
ZO-1	XM_013993251.1	F: GCATGATGATCGTCTGTCCTACC<br>R: CCGCCTTCTGTATCTGTGTCTTC	108
TGF-β1	NM_214015.2	F: AAAGCGGCAACCAAATCTATGA<br>R: GCTGAGGTAGCGCCAGGAAT	206
Smad2	NM_001256148.1	F: CACCGTAGATGGCTTCACAGA<br>R: CGCACTCCTCTTCCTATATGTCTT	120
Smad3	NM_214137.1	F: ACGACTACAGCCATTCCATCC<br>R: CTCTCCATCTTCACTCAGGTAGC	110
Smad4	NM_21407.1	F: CAGGACAGCACAGAATGGATT<br>R: GGTGAGGCAAATTAGGTGGGTATG	111
Smad7	NM_001244175.1	F: TACTGGGAGGAGAAGACGAGAGTG<br>R: TGGCTGACTTGATGAAGATGGG	241

ZO-1, zonula occludens-1; TGF-β1, transforming growth factor-β1.

Supplemental Methods

Immunohistochemistry Analysis

Sections were processed in accordance with the standard IHC protocols. After dewaxing, rehydration and antigen retrieval was performed by microwaving for 10 min at full power in sodium citrate buffer (0.01 mol/L, pH = 6.0). The sections were subsequently treated with 10% hydrogen peroxide (H2O2) for 1.5 h to deactivate endogenous peroxidase activity and incubated in 10% fetal bovine serum (11011-8611, TIANHANG, Huzhou, China) for 1 h to block nonspecific binding. The immunohistochemical analysis was performed using a commercial kit (Polink-2 plus® Polymer HRP Detection system for rabbit primary antibody, PV-9001, ZSGB-BIO, Beijing, China) according to the manufacturer's instructions. Briefly, after washing with phosphate-buffered saline (PBS), the above prepared sections were incubated with rabbit anti-TFF3 antibody (1:100, bs-0535R, Beijing, China), at 4 °C. The sections were washed with PBS the following day and were subsequently incubated in polymer helper for 1 h at 37 °C followed by Polink-2 plus polymer HRP anti-rabbit at 37 °C for 1 h. After this incubation, the sections were washed with PBS, followed by immersion in diaminobenzidine tetrachloride (DAB) using a kit (DAB kit, TIANGEN PA110, Beijing, China) for 1~3 min to detect immunostaining. The sections were then dehydrated, sealed in clear resin, mounted, and observed microscopically for the localization of immunoreactive substances using a bright field of view.

Gene Expression

Total RNA was extracted from samples preserved in RNase-free 2-mL frozen tube using AG RNAex Pro (Accurate Biology, Changsha, China) according to manufacturer's instructions. The purity and concentration of the RNA was assessed using an Eppendorf Biophotometer (DS-11, Denovix, USA) at an absorbance ratio of 260/280 nm (values in the range 1.8–2.0 indicate a pure RNA sample). The integrity of RNA was verified by agarose gel electrophoresis. A kit (Evo M-MLV Mix Kit with gDNA Clean for qPCR, AG11728, Accurate Biology, Changsha, China) was used for clean of gDNA and reverse transcription of total RNA. For qRT-PCR, the total volume of the PCR reaction mixture was 20 μL, which contained SYBR® Green Premix Pro Taq HS qPCR Kit (AG11701, Accurate Biology,

Changsha, China). Each sample was analyzed in triplicates. The optimized qRT-PCR protocol included an initial denaturation step at 95 °C for 30 s, followed by 43 cycles at 95 °C for 5 s, 60 °C for 30 s, 95 °C for 10 s, 60 °C for 60 s and 97 °C for 1 s. The qRT-PCR reactions were performed on a LightCycler 96 (Roch, Switzerland). The relative amounts of relative mRNA were expressed and calculated as equal to $2^{-\Delta\Delta CT}$.

Western Blot Analysis

The total protein of cecum was extracted using the lysate instructions (Beyotime, Shanghai, China) and quantitated using a bicinchoninic acid (BCA) protein assay kit (Beyotime, Shanghai, China). The sample amount was 50 μg protein per sample. Proteins were separated using electrophoresis on polyacrylamide gels and were subsequently transferred to nitrocellulose membranes (Merck Milipore, USA). After trimming and washing with 3 times for 10 min, the membranes were incubated in western blocking solution (Beyotime, Shanghai, China), and then incubated with the following primary antibodies: β-Actin rabbit monoclonal antibody (1:1000; Beyotime, Shanghai, China), rabbit anti-TFF3 (1:500, bs-0535R, BIOSS, Beijing, China), rabbit anti-occludin antibody (1:500, bs-10011R, BIOSS, Beijing, China), rabbit anti-claudin 1 (1:500, bs-10008R, BIOSS, Beijing, China), rabbit anti-ZO-1 (1:500, bs-1329R, BIOSS, Beijing, China), rabbit anti-TGF-β1 (1:500, bs-0103R, BIOSS, Beijing, China), rabbit anti-Smad2 (1:500, bs-0718R, BIOSS, Beijing, China), rabbit anti-Phospho-Smad2 (Ser465 + Ser467) (1:500, bs-3419R, Beijing, BIOSS, China), rabbit anti-Smad3 (1:500, bs-3484R, BIOSS, Beijing, China), rabbit anti-Phospho-Smad3 (Ser423 + Ser425) (1:500, bs-3425R, BIOSS, Beijing, China), rabbit anti-Smad4 (1:500, bs-23966R, BIOSS, Beijing, China), and rabbit anti-Smad7 (1:500, bs-0566R, BIOSS, Beijing, China) at 4 °C overnight. Then the membranes were incubated with HRP-labeled Goat Anti-Rabbit IgG(H+L) (1:2000, Beyotime, Shanghai, China), diluted in secondary antibody dilution buffer (Beyotime, Shanghai, China) at 37 °C for 2 h. Then the membranes were immersed in a super sensitive ECL chemiluminescence reagent (BeyoECL Star, Beyotime, Shanghai, China), exposed to film using the FUSION FX7 (Vilber Lourmat, Paris, France) and analyzed using Fusion 16.0.9.0 software (Vilber Lourmat, Paris, France).
